# The Impact of Workplace Harassment on Health in a Working Cohort

**DOI:** 10.3389/fpsyg.2019.01181

**Published:** 2019-05-24

**Authors:** Sara Gale, Irina Mordukhovich, Sami Newlan, Eileen McNeely

**Affiliations:** ^1^Department of Environmental Health, Harvard T.H. Chan School of Public Health, Boston, MA, United States; ^2^Department of Epidemiology, Harvard T.H. Chan School of Public Health, Boston, MA, United States

**Keywords:** occupational health, sexual harassment, verbal abuse, depression, anxiety, sleep disturbance

## Abstract

**Background:** Workplace abuse, including sexual harassment, is frequently experienced worldwide and is related to adverse mental health outcomes, and injuries. Flight attendants are an understudied occupational group and are susceptible to harassment due to working in a feminized, client-facing occupation with few protections or sanctioned responses against aggressive behaviors.

**Objective:** We investigated the relationship between workplace abuse and health in a cohort of cabin crew. We also aimed to characterize perpetrator profiles.

**Methods:** We conducted our study among 4,459U.S. and Canada-based participants from the Harvard Flight Attendant Health Study using multivariate logistic regression. Our exposures of interest were episodes of workplace abuse in the past year. We evaluated several mental and physical health outcomes, including depression, fatigue, musculoskeletal injuries, and general workplace injuries.

**Results:** We report that exposures to verbal abuse, sexual harassment, and sexual assault are common among cabin crew, with 63, 26, and 2% of respondents, respectively, reporting harassment in the past year alone. Workplace abuse was associated with depression, sleep disturbances, and musculoskeletal injuries among male and female crew, with a trend toward increasing odds ratios (ORs) given a higher frequency of events. For example, sexual harassment was related to an increased odds for depression (OR = 1.91, 95% confidence interval [CI]: 1.51–2.30), which increased in a dose response-like manner among women reporting harassment once (OR = 1.44, 95% CI: 0.93–1.95), 2–3 times (OR = 1.83, 95% CI: 1.29–2.38), and 4 or more times (OR = 4.12, 95% CI: 3.18–5.06). We found that passengers were the primary perpetrators of abuse.

**Conclusions:** Our study is the first to comprehensively characterize workplace abuse and harassment and its relation to health in a largely female customer-facing workforce. The strong associations with health outcomes observed in our study highlights the question of how workplace policies can be altered to mitigate prevalent abuses. Clinicians could also consider how jobs with high emotional labor demands may predispose people to adverse health outcomes, educate patients regarding their psychological/physical responses and coping strategies, and be aware of signs of distress in patients working in such occupations in order to direct them to the appropriate treatments and therapies.

## Introduction

Workplace harassment and abuse, especially against women, occur with great frequency worldwide (Krieger et al., 2006). Estimates suggest that as many as 50% of U.S. women experience sexual harassment during their working lives (Das, 2009), but only a minority report it (Feldblum and Lipnic, 2016). Studies indicate that workplace abuse and stress are related to poorer mental health, including sleep disorders, depression, anxiety, post-traumatic stress disorder and symptoms, and psychological distress (Gunnarsdottir et al., 2006; Nabe-Nielsen et al., 2016). This can be the case even for co-workers who are not directly victimized (Di Marco et al., 2016, 2018). Exposure to workplace stress has also been associated with increased musculoskeletal injuries and disorders and a higher cardiovascular risk score among flight logistic workers and flight attendants (Lee et al., 2008; Lecca et al., 2018).

The systems that are currently in place have proven insufficient to prevent workplace abuse (Fitzgerald, 1993; Okechukwu et al., 2014; Burke and Cooper, 2018). Victims are often left without support, within their job or from clinicians, while navigating the fallout of these experiences. Few studies have tracked the impact of workplace abuse on long-term health, although evidence suggests that sexual harassment early in the career has long-term effects on depressive symptoms, which in turn can affect quality of life, relationships, and professional attainment (Houle et al., 2011). The effects of harassment on workers can also in turn hurt organizations by affecting worker morale, productivity, absenteeism, turnover, organizational commitment, as well as the external reputation of the employer (McDonald et al., 2015).

Effective prevention rests on a detailed analysis of the current context of workplace harassment and abuse, including the characteristics of perpetrators, worker profiles (e.g., age, race, sexual orientation), and the timing of harassment. Prevention strategies include clearly stated company policies that provide workers and supervisors with proper training and sanctioned tools to respond to abuse and abusers (Fitzgerald, 1993; Okechukwu et al., 2014; McDonald et al., 2015).

Flight attendants are an understudied occupational group exposed to a wide range of biological and psychosocial stressors, including cosmic ionizing radiation at altitude, severe circadian rhythm disruption, chemical contaminants in the aircraft cabin, hypoxia, noise, heavy physical, and psychological job demands, and verbal and sexual harassment (Ballard et al., 2006; Griffiths and Powell, 2012). To our knowledge, our study is one of only a few to evaluate sexual harassment among flight attendants in relation to health and is the largest and most comprehensive study on this topic (Ballard et al., 2006; Gunnarsdottir et al., 2006). Flight attendants are a susceptible occupational group due to employment in a mostly female profession with high emotional labor demands (i.e., they are expected to suppress and regulate their emotional affect and responses according to employer and passenger expectations; Grandey and Melloy, 2017). Yet, no specific policies are in place for them to navigate abusive workplace interactions. Other professions share similar characteristics, and findings from our cohort may therefore be generalizable to a much wider range of occupational groups.

We aim to characterize the health impact of workplace abuse and harassment among workers, as well as characterize the prevalence of harassment and perpetrator profiles (supervisor, passenger, etc.), within a large ongoing cohort of cabin crew (McNeely et al., 2014, 2018). We hypothesized that passengers would be the most frequent perpetrators of workplace abuse against crew, and that verbal and sexual abuse occurring within the past year would be related to depression as well as sleep disturbances and fatigue over the past 2 weeks and to workplace accidents and musculoskeletal injuries over the past year, especially among those workers experiencing a higher frequency of abusive events.

## Methods

### FAHS Cohort Recruitment and Survey

#### Cohort Recruitment

Participants were enrolled in the second wave of the Harvard Flight Attendant Health Study (FAHS), an ongoing study established in 2007 with 4,011 participants (McNeely et al., 2014, 2018). For the 2014–2015 wave reported here, we recruited new and returning participants through several channels, including a hard copy survey mailed to the 2007 participants and an online survey launched in December 2014. We also conducted in-person recruitment at five U.S. airport hubs between December 2014 and June 2015, where we distributed postcards with the online survey URL and hardcopy surveys. Our campaign included email and flier announcements from local unions, as well as a study website and social media presence.

Current or former male and female U.S.- or Canada-based flight attendants were eligible to participate in the current survey, with no other eligibility criteria (beyond being an adult of at least 18 years of age, which is a requirement for being a flight attendant). In order to maximize the gender-stratified samples, no other exclusion criteria were used. We collected 1,642 surveys from returning participants, yielding a 40% response rate from the original cohort with valid addresses. While in 2007, the FAHS used paper surveys and found recruiting in-person at airport hubs to be most effective, in 2014 we switched to primarily online recruitment and questionnaires. We created a website for new and returning study participants to read about our research and to complete the questionnaire online as well as a social media campaign to provide participants with up-to-date news. In addition, we found that email blasts from flight attendant unions improved recruitment immensely. We continued passively collecting surveys until closing our online survey at the end of the sampling period.

Our mixed methods recruitment approach was similar to that used by recent high-profile studies, marking a shift toward accessible and adaptable online surveys formatted for smart phones and tablets, which give participants a secure, anonymous space to report on sensitive health topics, including sexual harassment (van Gelder et al., 2010). Our research was approved the Harvard T.H. Chan School of Public Health's Institutional Review Board. All participants provided their written informed consent prior to enrollment in the study.

#### Survey

Our survey was developed from numerous focus groups with flight attendants (which were arranged by a union to provide insight about the study questions from a larger Federal Aviation Administration study), and from validated questions about health outcomes and symptomology, work experiences and exposures, and demographic factors and personal characteristics taken from established surveys such as the Job Content Questionnaire and the National Health and Nutrition Examination Survey (Karasek et al., 1998; National Health and Nutrition Examination Survey, 2013-2014). Specifically, the questions we used regarding workplace abuse and harassment were the same as those used by Nurses' Health Study (as described in more detail below); these and other questions from national surveys were selected in order to facilitate comparisons across study populations (Bao et al., 2016). Participants were also asked to provide aviation employment history, including airlines, primary hubs, and dates of employment and leave. The survey includes 3 sections about each participant's job, personal characteristics, and health, with a total of approximately 100 questions (which varied depending on respondents' answers to questions with branching logic). The final survey instrument was tested in a sample of flight attendants before use in the 2007 study, and we further updated the survey in 2014 to account for feedback from participants enrolled in the original 2007 study and to refine our research interests based on earlier findings. For example, we included new questions on workplace harassment and as well as questions with finer detail for sleep outcomes and depression.

### Exposures and Outcomes

To measure exposures to workplace abuse in the past year, we used the following questions adapted from the Nurses' Health Study III, a longitudinal cohort study of U.S. nurses (Bao et al., 2016):

1. In the last 12 months, have you been sexually harassed at work (any type of unwelcome sexual behavior [words or actions] that creates a hostile work environment)?
2. In the last 12 months, have you been threatened or experienced verbal abuse at work (e.g., yelled at, shouted at, or sworn at)?
3. In the last 12 months, have you been sexually assaulted at work (someone used threat or force to engage in an unwanted sexual act)?

Because sexual harassment and workplace abuse tend to be vastly underreported through official channels, we used these self-reported measures of harassment and abuse in our study rather than drawing from organizational records or asking only about officially reported incidences of harassment (Feldblum and Lipnic, 2016).

We asked participants about depressive symptoms in the past 2 weeks using the validated Patient Health Questionnaire (PHQ)-9 scale, with depression based on a score of 10 or higher (Kroenke et al., 2001). The PHQ-9 is a brief, validated instrument which has been shown to be reliable for diagnosing depressive disorders, as well as for determining depressive symptom severity, and is shorter and more straightforward than previous comparable measures. It is important that this instrument is validated, short, and straightforward, as depressive disorders are commonly encountered in primary care settings with limited time for assessment and follow-up, and it facilitates our measuring depression and depression severity accurately within a comprehensive study survey (Kroenke et al., 2001). To examine associations with depression in our study, we dichotomized the depression variable using a threshold score of 10 (with 10 representing the lowest value for moderate depression). We also asked about sleep disturbances and fatigue symptoms in the past 2 weeks, and categorized responses into binary variables based on frequency of symptoms (with symptoms occurring “nearly every day” considered as a positive response). Finally, we queried participants about injuries or illnesses in the past 12 months that they considered to be work-related, and about specific musculoskeletal problems and injuries, including strains, sprains, joint pain, and fractures/contusions occurring in the past 12 months.

### Analytic Sample and Statistical Analysis

Our analytic sample includes 4,549 participants who worked as cabin crew within the last year. Retired flight attendants and those who did not work in a cabin within the previous year were excluded because they would not have experienced exposure to workplace harassment as a flight attendant in the in the time frame relevant to our study. There were no exclusions based on any other factors, such as gender, age, or seniority; this allowed us to maximize the sample of flight attendants we were able to reach. For each analysis, participants with non-missing data on the abuse exposure, health outcome, and adjusting covariates were drawn from this sample. Sample sizes differed across analyses and are shown in the tables. We calculated descriptive statistics for participant characteristics, as well as for the prevalence of verbal abuse, sexual harassment and sexual assault overall, by frequency of events in the past year, and by perpetrator type (supervisor/pilot, passenger, co-worker, and other—such as airport employees).

We then evaluated the association between verbal and sexual harassment or violence in the past 12 months (any vs. none) and depression, sleep disturbances and fatigue, work-related accidents and illnesses, and specific musculoskeletal conditions and injuries in a cross-sectional analysis, using multivariate logistic regression and adjusting for the following potential confounders: age (continuous), race (White vs. other), Hispanic ethnicity (yes/no), current smoking status (smoker/non-smoker), and job tenure as a flight attendant (continuous). All analyses were gender-stratified. We also conducted analyses stratified according to frequency of each type of event during the past year (occurring 1, 2–3, or 4+ times). Analyses were completed using STATA software (StataCorp, College Station, Texas).

## Results

We report characteristics of the study sample in Table 1. Participants presented with a median age of 50 years and a median job tenure of 18 years. Almost 80% of our cohort was female and 9% were current smokers. Over 90% had completed at least some college; 88% were American and 12% were Canadian.

**Table 1:** Characteristics of the Harvard Flight Attendant Health Study cohort (2014–2015).

**Participant characteristics**	**Counts or Medians (total *n* = 4,549)**	**Percent or standard deviation**
**Sex**		
Female	3,612	79.4%
Male	937	20.6%
Age (years)	50	11.27
**Race/ethnicity**^**a**^		
Hispanic, Latino	411	9%
Asian	214	4.7%
Black	179	3.9%
Native American	22	0.5%
White	3,665	80.6%
Other	282	6.2%
Missing/Don't Know	187	4.1%
**Education**		
High school or less	444	9.8%
Some college or Associate's degree	2,049	45%
Bachelor's degree	1,559	34.3%
Graduate school	361	7.9%
Missing	179	3.9%
Current smoker (yes)	402	8.8%
Job tenure (median years)	18	11.51
**Survey type**		
Canadian	566	12.4%
U.S.	3,983	87.6%

a *Categories are not mutually exclusive*.

We report the prevalence of workplace abuse experienced in the past year (subdivided into categories of verbal abuse, sexual harassment, and sexual assault), overall, by frequency of events and by perpetrator type (passenger, co-worker, etc.) in Table 2. In the past year alone, 63% of cabin crew experienced verbal abuse, 26% experienced sexual harassment, and 2% had been sexually assaulted. Many participants reported repeated abusive events. The majority of those experiencing verbal abuse and/or sexual harassment had two or more such experiences (per category) in the past year and a non-trivial percent experienced four or more events, though this was not the case for sexual assault. Passengers were the most common source of verbal abuse (89.6%), sexual harassment (68.7%), and sexual assault (46%) directed at crew (Table 2).

**Table 2:** The prevalence of workplace harassment and assault in the Harvard Flight Attendant Health Study (2014–2015), presented overall, by frequency of events, and by perpetrator type.

**Survey question**	**Sample size (total *n*= 4549)**	**Percentage (%)**
**Threatened or experienced verbal abuse at work in the**		
**past year**		
No	1,603	36.7
Yes	2,769	63.3
1 time	918	21.0
2–3 times	1,251	28.6
4 or more times	600	13.7
**Source of threat or verbal abuse, if reported**^**a**^		
Supervisor or pilot	285	10.3
Co-worker	474	17.1
Passenger	2,482	89.6
Others (e.g., airport employee)	367	13.3
**Sexually harassed at work in the past year**		
No	3,261	73.7
Yes	1,167	26.4
1 time	555	12.5
2–3 times	469	10.6
4 or more times	143	3.2
**Source of sexual harassment, if reported**^**a**^		
Supervisor or pilot	365	31.3
Co-worker	352	30.2
Passenger	802	68.7
Other	186	15.9
**Sexually assaulted at work in the past year**		
No	4,306	97.9
Yes	94	2.1
1 time	73	1.7
2–3 times	14	0.3
4 or more times	7	0.2
**Source of sexual assault, if reported**^**a**^		
Supervisor or pilot	23	24.5
Co-worker	24	25.5
Passenger	46	48.9
Other	21	22.3

a *Categories are not mutually exclusive. There could have been more than one source selected for the verbal abuse, harassment, and sexual assault. Percentages for the source categories are calculated with the total as the number of flight attendants who reported “Yes” to the workplace harassment question*.

We present gender-stratified results for associations between workplace abuse (the specific exposure variables of interest were verbal abuse, sexual harassment, and sexual assault) and physical and psychological health outcomes as represented by (1) depression assessed by a validated scale, (2) fatigue and sleep disturbance symptoms in the preceding 2 weeks, (3) work-related injuries in the past year, (4) and musculoskeletal injuries and conditions in the past year in Table 3. We report associations between all types of workplace abuse and physical and mental health outcomes. For example, among females, verbal abuse was positively related to depression (OR = 2.09, 95% CI: 1.74–2.45), work-related injury or illness (OR = 3.17, 95% CI: 2.35–4.26), sleep disturbance/fatigue (OR = 1.82, 95% CI: 1.43–2.31), musculoskeletal strain, sprain, and pain (OR = 1.62, 95% CI: 1.38–1.91), and fractures or contusions (OR = 1.72, 95% CI: 1.28–2.33). For sexual harassment, the corresponding effect estimates among females were OR = 1.91 for depression (95% CI: 1.52–2.30), OR = 3.48 for any workplace injury or illness (95% CI: 2.22–5.44), OR = 1.75 for sleep disturbance and fatigue (95% CI: 1.30–2.35), OR = 1.83 for musculoskeletal strain, sprain, and joint pain (95% CI: 1.52–2.21), and OR = 1.51 for fractures or contusions (95% CI: 1.13–2.02). Patterns were generally similar, though less precise, for male participants, and when evaluating sexual assault in relation to these outcomes, though verbal harassment was not related to sleep disturbances/fatigue or to fractures/contusions among men, and sexual harassment was likewise not related to fractures/contusions among men (Table 3).

**Table 3:** Associations between threats/verbal abuse, sexual harassment, and sexual assault with health outcomes in the Harvard Flight Attendant Health Study (2014–2015), adjusted for age, tenure, Hispanic ethnicity, white race, current smoking status.

**Threats or verbal abuse**	**Outcome**	**OR**	**95% CI**	***n* in model**
Females	Depression (from PHQ-9 scale)	2.09	(1.74, 2.45)	3,240
	Any injury/illness because of FA job	3.17	(2.35, 4.26)	3,373
	Sleep disturbances and fatigue	1.82	(1.43, 2.31)	3,160
	Musculoskeletal strain, sprain, joint aches, and pain	1.62	(1.38, 1.91)	3,160
	Musculoskeletal fracture or contusion	1.72	(1.28, 2.33)	3,160
Males	Depression (from PHQ-9 scale)	2.72	(1.93, 3.50)	861
	Any injury/illness because of FA job	3.20	(1.94, 5.27)	894
	Sleep disturbances & fatigue	0.91	(0.55, 1.50)	821
	Musculoskeletal strain, sprain, joint aches, and pain	1.56	(1.14, 2.14)	821
	Musculoskeletal fracture or contusion	0.93	(0.49, 1.77)	821
**Any sexual harassment**	**Outcome**	**OR**	**95% CI**	***n*** **in model**
Females	Depression (from PHQ-9 scale)	1.91	(1.52, 2.30)	3,277
	Any injury/illness because of FA job	3.48	(2.22, 5.44)	3,415
	Sleep disturbances and fatigue	1.75	(1.30, 2.35)	3,199
	Musculoskeletal strain, sprain, joint aches and pain	1.83	(1.52, 2.21)	3,199
	Musculoskeletal fracture or contusion	1.51	(1.13, 2.02)	3,199
Males	Depression (from PHQ-9 scale)	3.03	(2.03, 4.02)	868
	Any injury/illness because of FA job	9.46	(2.28, 39.32)	902
	Sleep disturbances and fatigue	4.71	(1.68, 13.20)	827
	Musculoskeletal strain, sprain, joint aches and pain	1.76	(1.18, 2.61)	827
	Musculoskeletal fracture or contusion	1.09	(0.49, 2.45)	827
**Any sexual assault**	**Outcome**	**OR**	**95% CI**	***n*** **in model**
Females	Depression (from PHQ-9 scale)	2.24	(1.03, 3.45)	3,257
	Any injury/illness because of FA job	4.56	(0.62, 33.01)	3,393
	Sleep disturbances and fatigue	1.76	(0.63, 4.87)	3,179
	Musculoskeletal strain, sprain, joint aches and pain	1.98	(1.08, 3.67)	3,179
	Musculoskeletal fracture or contusion	1.64	(0.77, 3.49)	3,179
Males	Depression (from PHQ-9 scale)	3.39	(0.40, 6.38)	865
	Any injury/illness because of FA job	1.77	(0.22, 14.16)	898
	Sleep disturbances and fatigue	–	–	–
	Musculoskeletal strain, sprain, joint aches, and pain	2.58	(0.70, 9.48)	823
	Musculoskeletal fracture or contusion	2.74	(0.54, 13.94)	823

*CI, confidence interval; FA, flight attendant; OR, odds ratio*.

**Figure 1:**
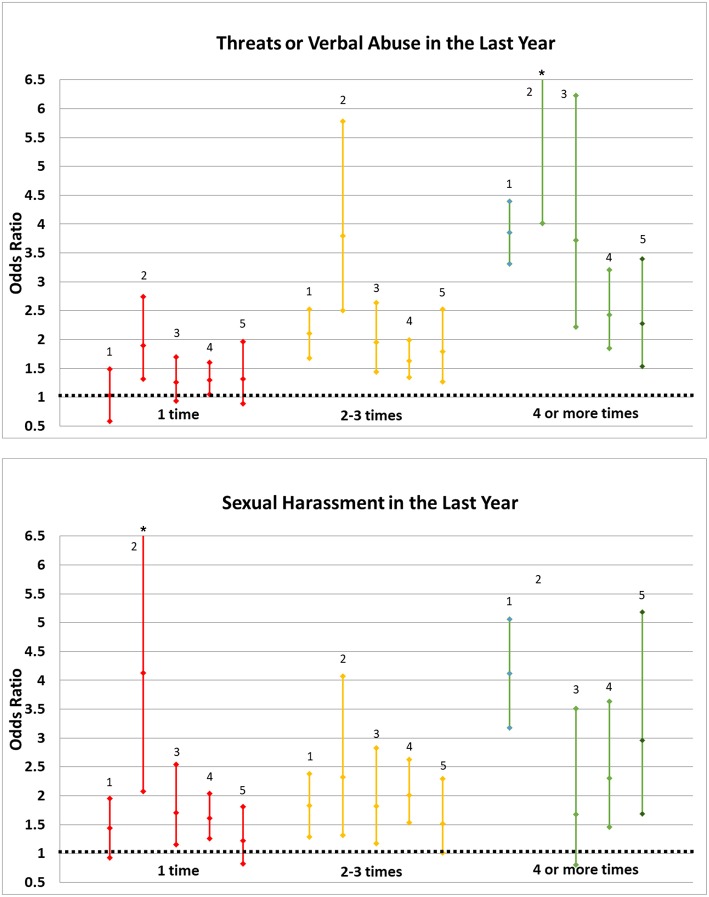
Odds ratios for health outcomes in relation to abuse and harassment episodes in the last year, Harvard Flight Attendant Health Study (2014–2015). 1: Depression 2: Any Work-Related Injury or Illness 3: Sleep Disturbance or Fatigue 4: Musculoskeletal Strain or Sprain, or Joint Aches/Pain 5: Musculoskeletal Fracture or Contusion. *Point estimates and confidence limits for work-related injury/illness among those with 4+ exposures to verbal abuse (OR = 9.89, 95% CI: 4.01, 24.36) and with 1 exposure to sexual harassment (OR = 4.13, 95% CI: 2.08, 8.11) are truncated. We were not able to calculate ORs and 95% CIs for work-related injury/illness among those with 4+ exposures to harassment.

We report associations between workplace abuse and health outcomes stratified by frequency of each type of event (1 time, 2–3 times, or 4 or more times in the past year) among females only (due to statistical power concerns) in Table 4 and show these results graphically in Figure 1. For example, we evaluated the association between verbal harassment and sleep disturbances/fatigue within strata of those experiencing 1 episode of verbal abuse, 2–3 episodes of verbal abuse, or 4 or more episodes of verbal abuse within the past year. We observed a pattern of stronger associations among women experiencing more events. For example, verbal abuse was not related to depression among women who had experienced one event in the past year (OR = 1.03, 95% CI: 0.58–1.49) but was associated with depression among those experiencing 2–3 events (OR = 2.10, 95% CI: 1.68–2.52) and 4 or more events (OR = 3.85, 95% CI: 3.31–4.39). Similarly, ORs between sexual harassment and depression were 1.44 (95% CI: 0.93–1.95) among those experiencing one event, 1.83 (95% CI: 1.29–2.38) among those experiencing 2–3 events, and 4.12 (95% CI: 3.18–5.06) among those experiencing 4 or more events. However, an increasing number of sexual harassment events was not meaningfully associated with sleep disturbances/fatigue or with fractures/contusions. Sample size was generally too low to obtain reliable and precise corresponding estimates for sexual assault in our sample (Table 4).

**Table 4:** Adjusted odds of work-related health outcomes and depression among females according to frequency of harassment and assault in the Harvard Flight Attendant Health Study (2014–2015).

**Outcome**	**Never (Referent)**	**1 time OR (95% CI)**	**2–3 times OR (95% CI)**	**4 or more times OR (95% CI)**
**Any threats or verbal abuse**				
Depression (from the PHQ-9 scale)	1.0	1.03 (0.58, 1.49)	2.10 (1.68, 2.52)	3.85 (3.31, 4.39)
**Work-related outcomes**				
Injury/Illness	1.0	1.90 (1.32, 2.74)	3.80 (2.50, 5.78)	9.89 (4.01, 24.36)
Sleep and Fatigue	1.0	1.26 (0.94, 1.70)	1.95 (1.44, 2.64)	3.72 (2.22, 6.23)
Musculoskeletal strain, sprain, joint aches and pain	1.0	1.30 (1.05, 1.60)	1.63 (1.34, 1.99)	2.43 (1.85, 3.21)
Musculoskeletal fracture or contusion	1.0	1.32 (0.89, 1.96)	1.79 (1.27, 2.52)	2.28 (1.53, 3.40)
**Any sexual harassment**				
Depression (from the PHQ-9 scale)	1.0	1.44 (0.93, 1.95)	1.83 (1.29, 2.38)	4.12 (3.18, 5.06)
**Work-related outcomes**				
Injury/Illness	1.0	4.13 (2.08, 8.11)	2.32 (1.32, 4.07)	–
Sleep and Fatigue	1.0	1.71 (1.15, 2.54)	1.82 (1.17, 2.83)	1.68 (0.80, 3.51)
Musculoskeletal strain, sprain, joint aches, and pain	1.0	1.61 (1.26, 2.04)	2.01 (1.53, 2.63)	2.30 (1.46, 3.63)
Musculoskeletal fracture or contusion	1.0	1.22 (0.82, 1.81)	1.52 (1.01, 2.29)	2.96 (1.69, 5.18)
**Any sexual assault**				
Depression (from the PHQ-9 scale)	1.0	1.72 (0.36, 3.08)	5.06 (2.08, 8.04)	1.18 (−4.53, 6.88)
**Work-related outcomes**				
Injury/Illness	1.0	3.53 (0.49, 25.70)	–	–
Sleep and Fatigue	1.0	1.35 (0.49, 3.78)	–	–
Musculoskeletal strain, sprain, joint aches and pain	1.0	2.08 (1.04, 4.17)	1.99 (0.42, 9.25)	1.05 (0.09, 11.97)
Musculoskeletal fracture or contusion	1.0	1.83 (0.81, 4.13)	1.16 (0.15, 9.11)	–

*CI, confidence interval; OR, odds ratio*.

## Discussion

Consistent with previous studies reporting reduced physical and psychological wellbeing in relation to workplace abuse (Ballard et al., 2006; Gunnarsdottir et al., 2006; Lee et al., 2008), we observed strong associations between all types of abuse and depression, sleep disturbances/fatigue, workplace injuries, and musculoskeletal conditions among cabin crew. Associations were generally strongest among those experiencing abuse with greater frequency in the past year. Our study confirms previous findings and extends the sparse literature on this topic in terms of types of abuse investigated, health outcomes considered, statistical power, and the inclusion of male cabin crew, showing associations between workplace abuse and health outcomes similar to those seen in women. This report informs future research directions and workplace policy considerations regarding the health, safety, and well-being of this understudied group of workers, as well as other service and health professionals with similar work environments and subject to similar workplace expectations.

To date, flight attendants are often not provided with sufficient training and tools to manage abusive interactions with passengers and are particularly susceptible to harassment due to employment in a profession that is primarily female and has been sexualized in popular culture. Flight attendants also contend with increased stress due to heightened onboard security since September 11, 2001, understaffing, increased passenger densities, and an increasing prevalence of distracted yet connected passengers with portable electronic devices that enable the capture and broadcast of onboard interactions through social media. As of 2018, the FAA Reauthorization Act requires each airline to have sexual misconduct policies and procedures in place, calls for the establishment of a National In-flight Sexual Misconduct Task Force, and mandates the Attorney General, in coordination with other Federal agencies, to start a reporting process for sexual misconduct (these changes will occur within a period of up to 2 years). The specific policies and procedures which will be chosen, as well as their practical enforcement and implementation, are crucial to the success of this endeavor, and as of now no policies are set to be in place to protect against verbal harassment of crew. We note also that a workplace culture that tolerates abuse as the status quo and fails to protect workers who are victims of abuse may be more likely to persist in the context of a global economic crisis, since workers may be especially fearful of losing their employment or job standing due to retaliation for speaking up, and because of concomitant stress and reduced psychological and physical health (Giorgi et al., 2015; Mucci et al., 2016).

Our findings are consistent with studies of workplace abuse across a range of professions, which consistently report that people experiencing workplace sexual harassment and other forms of abuse have higher rates of psychological distress, adverse mental health outcomes, some adverse physical health outcomes, and negative job-related consequences, including when these associations have been evaluated prospectively in a limited number of publications to date (Keashly, 1997; McDonald, 2012; Nielsen and Einarsen, 2012). Only two prior studies of sexual harassment and health specifically among flight attendants were conducted in Europe over 10 years ago. Our study extends this sparse literature to the U.S. and Canada, which employ over 100,000 flight attendants and have different cultures in many respects (Bureau of Labor Statistics, 2018). We also present updated findings in light of a changing cross-cultural work environment for cabin crew, including an older and more diverse work force, increasingly rigorous job demands and customer service expectations, an updated fleet (often meaning more passengers per plane), and more aggressive and entitled passengers (American Customer Service Index, 2018). A finding of note is the high prevalence of abuse experienced in the past year alone, at rates much higher than in a European cohort from over a decade ago (Ballard et al., 2006), but more consistent with a recent survey querying about the prevalence sexual harassment experienced by members of the Association of Flight Attendants (Association of Flight Attendants-CWA, 2018). These higher rates may be due to true increased prevalence or to differences in cultural perceptions of harassment, as both studies evaluated self-reported harassment (Gunnarsdottir et al., 2006).

A study of self-reported well-being among Icelandic flight attendants, nurses, and teachers, all of whom work in mostly female professions with service-oriented and protective roles and high emotional labor demands, found that repeated harassment, bullying, violence, and threats were related to reduced physical and psychological well-being within all groups, though it is difficult to quantitatively compare their findings to ours as the researchers reported neither odds/risk ratios nor associated confidence intervals for these associations (Gunnarsdottir et al., 2006). A large study among Italian flight attendants likewise found that harassment by passengers was related to self-reported fair to poor health, with an odds ratio of 2.83 (95% CI: 1.30–6.18), which is comparable to our findings (Ballard et al., 2006). This study did not, however, find evidence of associations between sexual harassment and current psychological distress (Ballard et al., 2006). We extend this research by focusing on specific diagnoses and symptomology, evaluating health effects of assault, and including male crew. By far the most common perpetrators of all types of workplace abuse against cabin crew were passengers, though supervisors and pilots, co-workers, and others (i.e., airport employees) composed a sizeable minority of abusers as well. This is consistent with figures reported for health care workers in largely female professions, who are most likely to be harassed by patients (Park et al., 2015).

Previous studies comparing the health of flight attendants to that of the general population report an increased prevalence of sleep disorders, fatigue, and depression among crew (McNeely et al., 2014, 2018). Female flight attendants are more likely to die of suicide than the general population (Ballard et al., 2002), and work as a flight attendant is linked to alcohol abuse (McNeely et al., 2018). This raises the question of to what extent stressful and traumatic interpersonal interactions influence health among cabin crew compared to other occupational factors, including shift work, long hours, separations from family, insufficient rest periods between flights, lack of institutional support, flight attendants' role as first responders (including possible trauma around crashes and terrorist attacks), social isolation, and inadequate availability of nutritious food at work (Griffiths and Powell, 2012).

Limitations of our study include its cross-sectional design, which precludes inferences about causality, though our use of structured questionnaires aims to minimize this bias. It is possible that people with mental health conditions are more vulnerable to experiencing abuse or to perceiving ambiguous interactions in a negative light, and the direction of causality is unclear. It is reassuring that our results are consistent with prospective studies of abuse and health outcomes (McDonald, 2012; Nielsen and Einarsen, 2012), such as a large study reporting that, among women, workplace sexual harassment at baseline was related to subsequent psychological distress, but psychological distress at baseline was not related to later experiencing harassment (Nielsen and Einarsen, 2012). These results were reversed for men, however, for whom psychological distress at baseline predicted experiencing sexual harassment by the time of follow-up (Nielsen and Einarsen, 2012). We note that even if people with mental health conditions are more likely to be abused, they could still experience worsened mental health as a result of these experiences. Although all data were collected simultaneously, we assessed depression and sleep disturbances during the previous 2 weeks, whereas we asked about abuse over the past year, thereby reducing the likelihood of reverse causality within the depression and sleep questions.

We note that health outcomes were self-reported, and validation through medical records was not possible due to the associated scope and cost. However, sensitivity and specificity are generally found to be moderate to high for musculoskeletal disorders and depression diagnoses (Picavet and Hazes, 2003; Sanchez-Villegas et al., 2008). Another potential limitation of our study was recruitment from a mix of company rosters, on-site airport recruitment campaigns, and an online/social media presence. This strategy may contribute to selection bias, as volunteer participants may be self-selecting relative to those recruited using a randomized approach and may differ in terms of socioeconomic status, attitude toward research, and/or other factors related to health or ability to complete surveys, as discussed in a recent analysis regarding online recruitment in the Heart eHealth Study (Guo et al., 2017). While it is unclear whether this self-selection would lead to disparate enrollment with regard to both the abuse exposures and health outcomes, differential missing data could contribute to selection bias if participants experiencing more frequent abuse and worse health outcomes omitted their responses to those items. However, we note that studies report that while online recruitment may lead to selection bias on a variety of factors, such as gender and marital status, it is much less likely to affect internal (rather than external) validity of exposure-outcome associations (Guo et al., 2017). This is likely to be especially true in a relatively homogenous workforce than in a general population study recruited online. It is also important to note than an online recruitment strategy has many advantages in terms of efficiency, reliability of data collection and coding, and the ability to reach a wider range of potential study participants (Guo et al., 2017).

Our study may have attracted a disproportionate number of flight attendants with psychological or physical health concerns, leading to detection bias, as flight attendants with worse health or exhibiting more psychological distress may be more motivated to participate in an epidemiological study of flight attendant health. However, it is reassuring that our results are consistent with previous studies that recruited participants uses more randomized approaches (McDonald, 2012; Nielsen and Einarsen, 2012). Also, it is reassuring that the gender distribution in our study is similar to the distribution within three prominent U.S. airlines, provided to us by their professional flight attendant union (data not shown).

An additional limitation of our study was insufficient statistical power to evaluate some associations among male participants or for assault, which occurred with much less frequency than harassment. Finally, we did not have sufficient power to evaluate health effect estimates by perpetrator type.

Strengths of our study include access to the resources of a large cohort of cabin crew with a wealth of information on multiple health outcomes, work experiences and exposures, and potential confounders. In addition, online questionnaires are an increasingly popular option in epidemiologic research, including in high profile studies such as the Millennium Cohort and the Nurses' Health Study (van Gelder et al., 2010; Bao et al., 2016). This mode of data collection allows for validation checks, personalized questions, convenience, and accessibility to participants, and equal or better validity compared to printed questionnaires (Guo et al., 2017).

## Conclusions

We report associations between workplace abuse and depression, sleep disorders, fatigue, and musculoskeletal injuries among a large cohort of workers. Our findings have implications for the health of cabin crew and other health and service professionals, as well as for worker productivity. Depression, fatigue and musculoskeletal injuries are related to reduced productivity and job performance, lower organizational commitment, increased absence from work, and early retirement (Hardy et al., 2003; Karpansalo et al., 2004). Our findings may also be applicable to passengers, who may be subject to harassment by the same perpetrators that abuse flight crew. Future studies are needed to replicate our findings and to evaluate these associations prospectively, as there are few longitudinal studies of the health effects of workplace abuse that would allow researchers to evaluate the direction of causality. Longitudinal studies should also evaluate associations between workplace abuse/harassment and work-related outcomes such as days of lost work due to psychological distress or sick leave or reduced organizational commitment. Future high-powered studies should evaluate whether associations between workplace abuse/harassment and health outcomes differ according to perpetrator characteristics.

The strong associations observed in our study and other research, as well as the high prevalence of reported abuse among cabin crew, highlights the question of how workplace policies can be altered to mitigate these prevalent abuses against crew and possibly fellow passengers. For example, protocols already exist for properly training supervisors and workers in ways to handle inappropriate behaviors, for prevention and remediation of workplace abuse, and in what specifically constitutes workplace abuse (McDonald et al., 2015). Specifically, conveying that harassment is a community (rather than individual) concern, encouraging and providing multiple channels for workers to seek advice and support (including in “gray area” situations), rewarding those that take appropriate (neutral and objective) action and disincentivizing those that retaliate against the complainant, clearly stated penalties for violations and abuses, universal training across the organization as well as specific training for managers or those in positions of power, and openly and visibly stating that workplace harassment and abuse will not be tolerated are all strategies strongly supported by the literature (McDonald et al., 2015).

Clinicians could also consider how jobs with high emotional labor demands may predispose people to adverse health outcomes from maltreatment, educate their patients or clients regarding their psychological or physical responses as well as on coping and response strategies (e.g., minimizing focus on the abuser in order to regain a sense of control), and be aware of signs of distress in patients working in such occupations (McDonald et al., 2015). Importantly, clinicians should also be prepared to refer patients to appropriate therapies and treatments following disclosure or signs of psychological distress.

## Ethics Statement

Our study was approved by the Institutional Review Board at the Harvard T.H. Chan School of Public Health. All participants provided their written informed consent prior to participation in our survey-based research.

## Author Contributions

SG designed and conducted statistical analyses for this study, wrote much of the manuscript, and oversaw many aspects of building and maintaining the Flight Attendant Health Study cohort and questionnaire, including the questions central to this analysis. IM interpreted the findings of the study and wrote much of the manuscript. SN was involved in study design and interpretation and statistical analysis, as well as lending her expertise regarding the sexual harassment epidemiology literature. EM helped design and interpret findings for the study and is the founder and Principal Investigator of the Flight Attendant Health Study. All authors reviewed the manuscript prior to submission to *Frontiers in Psychology*.

Anmol Chaddha contributed to the manuscript by aiding in statistical analyses, and Anthony Brown was instrumental in the recruitment efforts for Wave 2 of the Flight Attendant Health Study.

### Conflict of Interest Statement

The authors declare that the research was conducted in the absence of any commercial or financial relationships that could be construed as a potential conflict of interest.
